# The Crosstalk of the Salicylic Acid and Jasmonic Acid Signaling Pathways Contributed to Different Resistance to Phytoplasma Infection Between the Two Genotypes in Chinese Jujube

**DOI:** 10.3389/fmicb.2022.800762

**Published:** 2022-03-18

**Authors:** Lixin Wang, Shiyan Liu, Mengjiao Gao, Lihu Wang, Linxia Wang, Yunjie Wang, Li Dai, Jin Zhao, Mengjun Liu, Zhiguo Liu

**Affiliations:** ^1^College of Horticulture, Hebei Agricultural University, Baoding, China; ^2^Research Center of Chinese Jujube, Hebei Agricultural University, Baoding, China; ^3^School of Landscape and Ecological Engineering, Hebei University of Engineering, Handan, China; ^4^College of Life Science, Hebei Agricultural University, Baoding, China

**Keywords:** Chinese jujube, phytoplasma, salicylic acid, jasmonic acid, ROS, signaling

## Abstract

Jujube witches’ broom disease (JWB), one of the most serious phytoplasma diseases, usually results in the destruction of Chinese jujube (*Ziziphus jujuba* Mill.). Although most jujube cultivars are sensitive to JWB, we found a few genotypes that are highly resistant to JWB. However, the molecular mechanism of phytoplasma resistance has seldom been studied. Here, we used Chinese jujube “T13,” which has strong resistance to JWB, and a typical susceptible cultivar, “Pozao” (“PZ”), as materials to perform comparative transcriptome, hormone, and regulation analyses. After phytoplasma infection, the differential expression genes (DEGs) were detected at all three growth phases (S1, S2, and S3) in “PZ,” but DEGs were detected only at the first growth phase in “T13.” Meanwhile, no phytoplasma was detected, and the symptoms especially witches’ broom caused by JWB were not observed at the last two growth phases (S2 and S3) in “T13.” Protein–protein interaction analysis also showed that the key genes were mainly involved in hormone and reactive oxygen species (ROS) signaling. In addition, during the recovered growth phase in “T13” from S1 to S2, the level of hydrogen peroxide (H_2_O_2_) was significantly increased and then decreased from S2 to S3. Moreover, jasmonic acid (JA) was significantly accumulated in “PZ” diseased plants, especially at the S2 phase and at the S2 phase in “T13,” while the content of salicylic acid (SA) decreased significantly at the S2 phase of “T13” compared to that in “PZ.” The changes in H_2_O_2_ and JA or SA were consistent with the changes in their key synthesis genes in the transcriptome data. Finally, exogenous application of an SA inhibitor [1-aminobenzotriazole (ABT)] rescued witches’ broom symptoms, while the contents of both JA and MeJA increased after ABT treatment compared to the control, demonstrating that exogenous application of an SA inhibitor rescued the symptoms of jujube after phytoplasma infection by decreasing the contents of SA and increasing the contents of JA and MeJA. Collectively, our study provides a new perspective on the transcriptional changes of Chinese jujube in response to JWB and novel insights that the crosstalk of JA and SA signaling communicated together to contribute to “T13” JWB resistance.

## Introduction

Phytoplasmas belonging to the class Mollicutes (Bertaccini and Lee, 2018) are cell wall-less parasites that cause destructive bacterial diseases that have been identified worldwide. Phytoplasma disease results in significant economic loss in many crop plants, fruit trees, ornamental plants, timber, and shade trees (Cousin, 1995; Bertaccini et al., 2014). Phytoplasmas are restricted to the phloem tissues in the plant host and induce typical symptoms, such as phyllody, witches’ broom, virescence, dwarfism, sterility of floral organs, wrinkled leaves, and yellowing (Liu et al., 2010). However, the special structure of phytoplasmas poses a challenge to cultivate them in media in the lab, and the distribution of phytoplasmas is irregular in host plants, greatly limiting the study of the molecular mechanism by which plants respond or resist phytoplasma infection.

Plants have evolved many defense mechanisms in response to pathogen attack. Among them, the plant immune system is activated by signaling transduction networks, such as calcium (Ca^2+^), reactive oxygen species (ROS), and hormones. First, phloem sieve tubes are the main location of phytoplasmas, and sieve elements have important functions in the propagation of phytoplasmas (van Bel and Musetti, 2019). Studies have shown that phytoplasma infection can modify the activities of Ca^2+^-permeable channels to regulate Ca^2+^ signaling in sieve elements (Musetti et al., 2013), demonstrating that Ca^2+^ might be one of the early signals in response to phytoplasma infection. Second, the generation of ROS is the most important early signaling molecule in response to pathogen attack. Higher production of ROS could damage plant cells, but rapidly moving ROS and lower contents of ROS could activate phytoalexins and pathogenesis-related genes to suppress pathogen development (Gao et al., 2018). For example, respiratory burst oxidase homolog D (RbohD)-dependent ROS accumulation can stimulate autophagosome formation and limit hypersensitive resistance (HR)-related cell death (Liu et al., 2015). Within phytoplasma infection, the rapid production of ROS in different plants has been elucidated (Musetti et al., 2005; Xue et al., 2020). Higher production of ROS was observed in phytoplasma-infected jujube leaves, and higher activities of glutathione S-transferase and peroxidase were detected in JWB-resistant cultivars, indicating that the roles of the antioxidant defense system are involved in phytoplasma attack (Xue et al., 2020). Increasing hydrogen peroxide (H_2_O_2_) content in phytoplasma-infected Napier grass was detected (Asudi et al., 2021). In addition, higher levels of H_2_O_2_ rather than superoxide were detected in phytoplasma-infected *Crassula argintea* (Dewir et al., 2016). Thus, ROS could be the second important class of signaling molecules to modulate the protective responses in plants to phytoplasma attack. The function of ROS signaling in response to phytoplasma infection is worth studying.

In addition to calcium and ROS, plant hormones could be the primary signaling molecules that function in the regulation of plant immunity (Verhage et al., 2010). The main hormones involved in plant immunity are abscisic acid (ABA), ethylene, salicylic acid (SA), and jasmonic acid (JA; Shigenaga et al., 2017; Dermastia, 2019; Koo et al., 2020). For example, ABA negatively regulates tomato in response to *Botrytis cinerea* by modulating the SA and JA pathways (Audenaert et al., 2002; Wan et al., 2021). Antagonistic crosstalk between JA and SA against pathogens was reported (Balbi and Devoto, 2008). SA was the first hormone that was reported as an elicitor against tobacco mosaic virus in tobacco (White, 1979). JA is another important plant hormone that is derived from the lipoxygenase pathway from the α-linolenic acid of chloroplast membranes. When grapevine was infected by “*Ca. P. solani*,” most JA biosynthetic genes were upregulated, especially the *VvLOX* genes, and JA-responsive *PR3/PR4* were significantly induced in the leaves of grapevine when it recovered from *bois noir*. In addition, exogenous application of SA could induce cucumber plants to resist powdery mildew disease (Koo et al., 2020). Besides JA, the contents of free and total SA were significantly induced in the main leaf veins during infections of grapevine with “*Ca. P. solani*” compared to uninfected samples (Paolacci et al., 2018), and a 26-fold increase in SA 2-O-glucopyranosyl was detected (Prezelj et al., 2016). In addition, SA-responsive genes involved in signal transduction, such as *VvEDS1* and *VvNPR1.2*, were significantly more highly expressed in symptomatic infected grapevines than in uninfected plants. *PR3* and *PR10* were upregulated in coconut after yellow decline phytoplasma infection (Nejat et al., 2015). Moreover, in response to phytoplasma infection, JA biosynthesis proteins were increased, and the key differentially expressed genes (DEGs) of JA biosynthesis or signaling transduction pathways were enriched, suggesting that JA might function during JWB recovery (Wang and Nick, 2017; Ye et al., 2017). However, JA and SA do not have synergistic effects under pathogen attack in the plant kingdom. Sometimes, an antagonistic effect between SA and JA signaling was observed. For example, the expression of JA-responsive marker genes *PLANT DEFENSIN 1.2* (*PDF1.2*) and *VEGETATIVE STORAGE PROTEIN 2* (*VSP2*) could be suppressed by exogenous application of SA (Spoel et al., 2007; Leon-Reyes et al., 2009). In addition, the expression of JA-responsive JA biosynthesis genes *LOX2*, *AOS*, *AOC2*, and *OPR3* could be downregulated by exogenous application of SA under pathogen or insect attack in *Arabidopsis thaliana* (Leon-Reyes et al., 2010). Phytoplasma-infected defective-mutant *Atseor1ko* plants also showed reduced phytoplasma contents with increased JA levels in the midribs of *Atseor1ko* plants at an early stage of infection (Bernardini et al., 2020). Thus, the antagonistic effect of SA on JA signaling might play a more important role in plant immunity than in individual immunity. In addition to JA and SA, the ratio between cytokines and auxin also plays an important role in the growth and development of Chinese jujube when it is infected by phytoplasma (Liu et al., 2010). However, due to the different research materials and methods, the involvement of plant hormones in phytoplasma–plant interactions is difficult to summarize, and there are no reports on how hormones regulate phytoplasma resistance in Chinese jujube.

Chinese jujube (*Ziziphus Jujuba* Mill.), originating from China, is known for resistance to most abiotic stresses. However, phytoplasma disease in Chinese jujube, also called Jujube Witches’ Broom Disease (JWB), destroys at least three to 5% of jujube trees in the world and affects final production (Liu et al., 2010, 2020a). Similar to other phytoplasmas, JWB phytoplasmas are phloem-limited and difficult to control; thus, cultivation of cultivars resistant to JWB could be an effective method to control its spread or to eliminate it in China. Through efforts over several years, we screened one genotype named “T13,” which could recover from JWB symptoms and be resistant to phytoplasma disease, and no phytoplasmas were identified in this genotype at the late growth stage, which had no effect on the yield. Finally, we identified this genotype as a JWB-resistant cultivar. Thus, we took the none resistant cultivar “Pozao” as a negative control to study the molecular mechanism how jujube “T13” was resistant to JWB. First, the concentration of phytoplasmas was detected in both materials at different growth phases, and then comparative transcriptome analysis and ROS, hormone detection, and SA inhibitor regulation analyses were performed, which provided important theoretical strategies for jujube JWB-resistant breeding.

## Materials and Methods

### Plant Materials and Phytoplasma Quantification

Two Chinese jujube cultivars grown at the Fuping Experimental Station of Chinese Jujube, Hebei Agricultural University, were used in the current study. *Ziziphus jujuba* Mill. “Pozao” (“PZ”) is a JWB-susceptible cultivar, and *Ziziphus jujuba* Mill. “T13” is a JWB-resistant cultivar. The scions from both healthy cultivars were grafted onto JWB-diseased rootstocks in May, as described by Xue et al. (2020), which were named “PZ_D” and “T13_D,” where D stands for disease. The scions grafted on healthy rootstocks were analyzed as a negative control and named “PZ_H” and “T13_H,” H meaning healthy. All experimental trees were cultivated under natural environmental conditions (Liu et al., 2020b). After grafting to the first time of sample collection, the scions began sprouting, spreading leaves, and grew up. On the 15th of June, witches’ broom leaves (shoots with small leaves, maximum severity) were observed, and shoots and leaf samples were collected from these four samples with at least three replicates from three different trees. The first sample collection was called growth phase “S1.” Then, we collected the samples on the 30th of June (growth phase “S2”) and on the 15th of July (growth phase “S3”). In the flowing contents, especially at the part of result, PZ S1D or T13 S1D represented the samples are from the S1 growth phase of diseased “PZ” and “T13,” respectively. The same format, such as PZ S1H or T13 S1H, has the corresponding meanings. Shoots and leaf samples were taken to the lab in an ice box for photography, frozen rapidly in liquid nitrogen, and stored at −80°C pending RNA isolation, quantitative real-time PCR (qRT–PCR) analyses, ROS production, and related enzyme activity and hormone content analysis.

Phytoplasma in all of the samples was quantified by qRT–PCR analysis of the *thymidylate kinase* gene (*TMK*, KC493615). The differential expression of *ZjTMK* in different phytoplasma symptom tissues could reflect the contents of phytoplasma in corresponding tissue (Bu et al., 2016; Xue et al., 2018).

### Measurement of MDA, H_2_O_2,_ and Activity of SOD, POD, and CAT

Malondialdehyde (MDA) and hydrogen peroxide (H_2_O_2_) detection: briefly, 1 g of leaf powder was extracted by 10 ml of trichloroacetic acid solution (100 g L^−1^) and centrifuged at 10,000 *g* for 20 min at 4°C, and the supernatant was kept to measure the MDA content. The MDA content was determined according to the MDA content assay kit method (Beijing Solarbio Science & Technology Co., Ltd., Beijing) and expressed in nM g^−1^ FW. To measure H_2_O_2_, 0.1 g leaf powder was extracted in 1 ml acetone reagent and incubated in an ice bath after centrifugation at 8,000 *g* for 20 min at 4°C. The supernatant was placed on ice to be tested by an H_2_O_2_ content assay kit (Beijing Solarbio Science & Technology Co., Ltd., Beijing).

Measurement of superoxide dismutase (SOD), peroxidase (POT), and catalase (CAT): briefly, 0.1 g leaf powder was extracted in 1 ml extraction reagents. After centrifugation at 8,000 *g* for 20 min at 4°C, the supernatant was placed on ice to be tested by the respective content assay kits (Beijing Solarbio Science & Technology Co., Ltd., Beijing).

### Detection of Phytohormones

The phytohormones were detected by ESI-HPLC–MS/MS analysis. Standard phytohormones were purchased from Sigma (China). Briefly, 0.5 g samples were ground in liquid nitrogen and put in glass test tubes, 10 times volume acetonitrile solution was added, and 4 μl internal standard mother liquor was added. The samples were extracted overnight at 4°C and centrifuged at 12,000 *g* for 5 min, and the supernatant was collected. The precipitate was added again with a 5-fold volume acetonitrile solution, and the supernatant was extracted twice and combined with previously described methods. Then, 35 mg C18 packing was added, and the solution was shaken for 30 s. Then, the solution was centrifuged at 10,000 *g* for 5 min, and the supernatant was retained, dried by nitrogen gas, dissolved with 400 μl methanol, and then passed through a 0.22 μM organic phase filter film. The liquid was transferred to a −20°C refrigerator to be further tested. A poroshell 120 SB-C18 column (2.1 × 150, 2.7 μm) was used for separation of phytohormones, and samples were eluted with solvent A (methanol/0.1% methyl acid) and solvent B (water/0.1% methyl acid). The mass spectrum parameters were ionization mode: scan type: MRM; air curtain gas: 15 psi; spray voltage: +4,000 V; atomizing gas pressure: 65 psi; auxiliary gas pressure: 70 psi; atomization temperature: 400°C. Finally, plant sample hormone content (ng/g) = detection concentration (ng/ml) × dilution volume (ml)/weighing mass (g).

### RNA Extraction and Illumina Sequencing

Total RNA from 18 samples was extracted from the leaves using a TRIzol® Reagent kit, and the RNA concentration and quality were determined by Nanodrop2000 and agarose gel electrophoresis. Then, Illumina sequencing was performed by Shanghai Majorbio Bio-pharm Biotechnology Co. (Shanghai, China). Briefly, 1 μg of total RNA was used to construct the RNA-seq transcriptome library using the TruSeq™ RNA sample preparation kit, from Illumina (San Diego, CA). The prepared paired-end RNA-seq transcriptome library was sequenced with an Illumina HiSeq xten/NovaSeq 6000 sequencer (2 × 150 bp read length; Gao et al., 2021).

### Transcriptome Data Analysis

The raw paired-end reads were trimmed and qualified by SeqPrep^1^ and Sickle^2^ according to default parameters. After filtering out adapters and low-quality sequences, the clean reads were separately mapped using TopHat^3^ (version 2.0.0; Langmead and Salzberg, 2012) to the jujube reference genome, which was downloaded from the NCBI website.^4^ The gene expression level was calculated according to the transcripts per million reads (TPM) using RSEM software^5^ (Li and Dewey, 2011). The Majorbio Cloud Platform^6^ was used to annotate genes to describe biological processes, molecular functions, and cellular components.

The analysis of differentially expressed genes (DEGs) was performed using the edgeR package. DEGs were identified according to value of *p* ≤ 0.05 and |log2 FC| ≥ 1. Gene Ontology (GO) and Kyoto Encyclopedia of Genes and Genomes (KEGG) enrichment analyses of DEGs were further implemented by Goatools^7^ and KOBAS^8^ software (Xie et al., 2011).

For the protein–protein interaction (PPI) analysis, the protein sequences of DEGs were used as queries to perform the prediction through the STRING database.^9^ The data were analyzed on the free online platform of the Majorbio Cloud Platform^6^ with default parameters. The results of data analysis were visualized by Cytoscape software (revision 3.6.1).

### qRT–PCR Analysis

qRT–PCR was performed on a Bio–Rad iQ™ 5 using TransStart Top Green qPCR SuperMix AQ131 (TransGen Biotech, China). The 20 μl reaction system contained 10 μl of 2 × SYBR Premix ExTaq™, 0.4 μl each of 10 μM primers, 1 μl diluted cDNA, and 8.2 μl ddH_2_O. The thermal profile was preincubated for 3 min at 94°C, followed by 40 cycles of 5 s at 94°C, 15 s at 55°C–63°C and 15 s at 72°C. Relative expression levels of selected DEGs were calculated by the 2^−ΔΔCt^ method (Livak and Schmittgen, 2001) using *ZjActin* as an endogenous control for normalization (Bu et al., 2016). The primer sequences of selected DEGs for qRT–PCR are shown in Supplementary Table S1.

### Treatment With Exogenous JA, SA, and Their Inhibitors

Tissue cultures of DH2 (showing witches’ broom symptoms) were treated with 100 μM MeJA and SA. Then, 100 μM JA synthesized inhibitor ibuprofen and SA synthesized inhibitor 1-aminobenzotriazole (ABT) were applied in DH2 tissue culture. After 1 month, the phenotype and content of JA were analyzed.

### Statistical Analyses

All data were analyzed by GraphPad Prism 8 software. Heatmaps were constructed by TBtools software (Chen et al., 2020). After conducting an analysis of variance, one-way Student’s *t*-test was used to evaluate the statistical significance.

## Results

### Resistant “T13” and Susceptible Genotype “PZ” Showed Significantly Different Phenotypic Changes After Phytoplasma Infection

**Figure 1:**
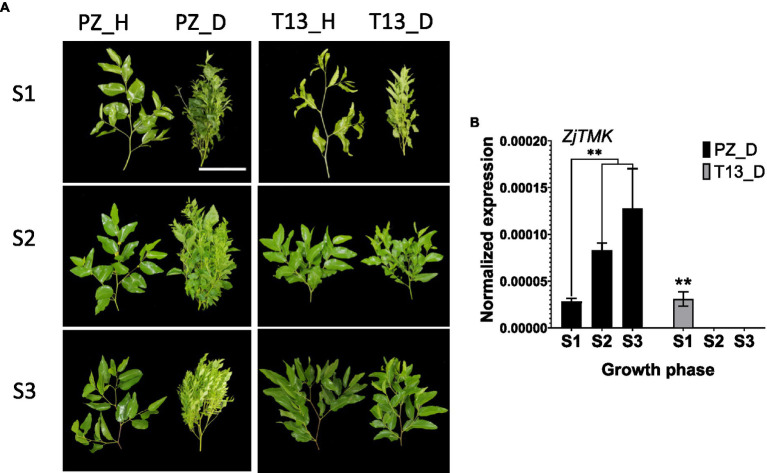
Phenotypes and expression of *ZjTMK* in “PZ” and “T13” shoots infected by phytoplasma. **(A)** Shoot phenotypes at three growth phases (S1, S2, and S3) of *Ziziphus jujuba* Mill. “Pozao” and “T13.” White bars are 10 cm. **(B)** The normalized expression level of *ZjTMK* in diseased *Ziziphus jujuba* Mill. “Pozao” and “T13” at three growth phases (S1, S2, and S3) in three independent replications, and the error bar represents the SE. “PZ_D” and “T13_D” represent diseased *Ziziphus jujuba* Mill. “Pozao” and “T13.” “PZ_H” and “T13_H” represent healthy *Ziziphus jujuba* Mill. “Pozao” and “T13.” *p < 0.05; and **p < 0.01.

To understand the differential molecular mechanism of phytoplasma infection between different jujube genotypes, the scions of healthy “PZ” (susceptible genotype) and “T13” (resistant genotype) were grafted on rootstocks with JWB to observe their phenotypic changes at three growth phases. As shown in Figure 1A, at growth phase S1, both resistant and susceptible genotypes showed witches’ broom symptoms, but the healthy control groups grew normally. Then, at growth phase S2, severe witches’ broom symptoms were observed in “PZ” but no symptoms were identified in “T13.” Furthermore, at growth phase S3, the witches’ broom symptoms were more severe in “PZ,” but “T13” grew normally and completely recovered and showed no difference compared to its healthy control (Figure 1A). To confirm whether the behavior of the resistant and susceptible genotypes infected by phytoplasma was consistent with the contents of phytoplasma changes, the expression level of *ZjTMK* was detected. As shown in Figure 1B, the expression level of *ZjTMK* increased at the three growth phases in “PZ”D, and its expression level could only be detected at growth phase S1, not in the other two growth phases, in “T13”_D. No expression level of *ZjTMK* could be detected in healthy plants of either resistant or susceptible genotypes. These results demonstrated that “T13” was more resistant to phytoplasma than “PZ,” and their phenotypes under phytoplasma infection were consistent with the phytoplasma contents in the leaves.

### Transcriptome Analysis Revealed Differences in Response to Phytoplasma Infection Between “PZ” and “T13”

As analyzed above, the symptoms at the S1 growth phases of “PZ” and “T13” in response to phytoplasma were completely different. However, the phenotype of “T13” represented the process by which it recovered from phytoplasma infection at S2 and S3 growth phases, indicating the resistance ability of “T13” to phytoplasma. According to the significantly differential behavior of “PZ” and “T13” in response to JWB, transcriptome analysis was performed to reveal which signaling pathway might contribute to their differential phytoplasma resistance. After sequencing, a total of 1,103,480,596 clean reads were obtained, and all of the Q30 base percentages were above 92.19%. In addition, the clean reads mapped to the Chinese jujube reference genome ranged from 80.73% to 89.67% (Supplementary Table S2), and 33,682 expressed genes were identified.

**Figure 2:**
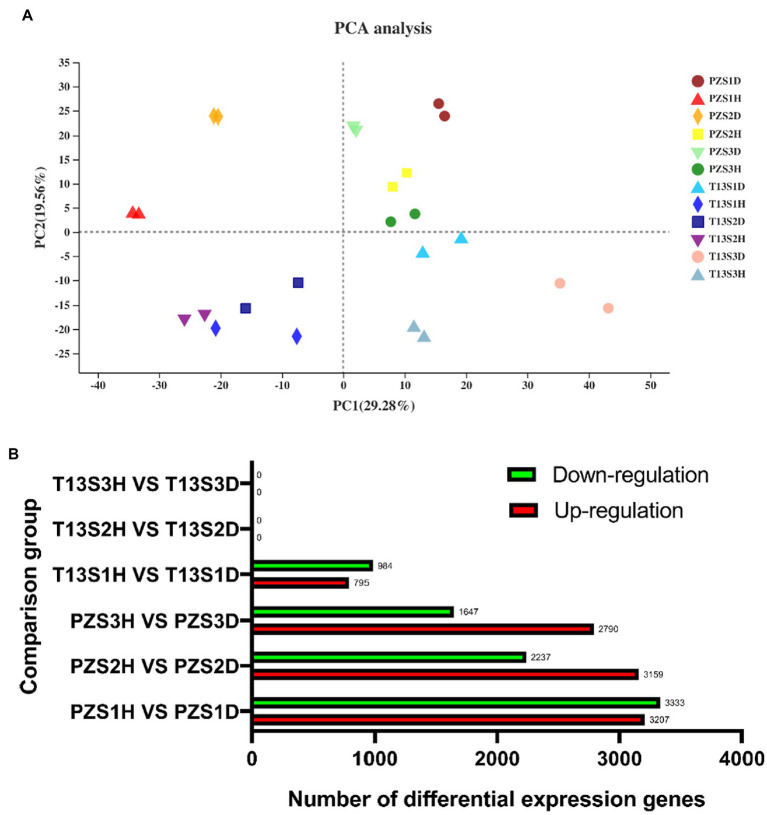
Principal component analysis (PCA) and count of differentially expressed genes (DEGS) analysis. **(A)** PCA of all samples in the 12 groups with two biological replicates. **(B)** Sum of up- and downregulated DEG counts at least twofold in six pairwise comparisons (T13S1H vs. T13S1D, T13S2H vs. T13S2D, T13S3H vs. T13S3D, PZS1H vs. PZS1D, PZS2H vs. PZS2D, and PZS3H vs. PZS3D) with adjusted *p* < 0.05.

Principal component analysis (PCA) showed that two sample replicates in each group were clustered together, demonstrating the reliability of the data for further analysis. Among these replicates, the two groups (PZ_D and PZ_H) of “PZ” in three growth phases (S1, S2, and S3) were distributed separately in different areas. However, only the two groups (T13_D and T13_H) of “T13” in growth phase S1 were distributed separately in different areas but not in growth phases S2 and S3, indicating that fewer transcriptome changes occurred during growth phases S2 and S3 of “T13” between healthy and phytoplasma-infected plants (Figure 2A).

Furthermore, the differentially expressed genes (DEGs) among six pairwise comparisons (T13S1H vs. T13S1D, T13S2H vs. T13S2D, T13S3H vs. T13S3D, PZS1H vs. PZS1D, PZS2H vs. PZS2D, and PZS3H vs. PZS3D) were analyzed. As shown in Figure 2B, different DEGs were identified in four groups (T13S1H vs. T13S1D, PZS1H vs. PZS1D, PZS2H vs. PZS2D, and PZS3H vs. PZS3D). Among these groups, the number of downregulated or upregulated DEGs increased from growth S1 to S3 in “PZ” and the number of DEGs was lower in T13S1H vs. T13S1D than in PZS1H vs. PZS1D. Interestingly, no DEGs were detected in Groups T13S2H vs. T13S2D and T13S3H vs. T13S3D. This finding is consistent with the phenotypic behavior of “T13” infected by phytoplasma, in which no witches’ broom symptoms were observed at these two growth phases (S2 and S3).

**Figure 3:**
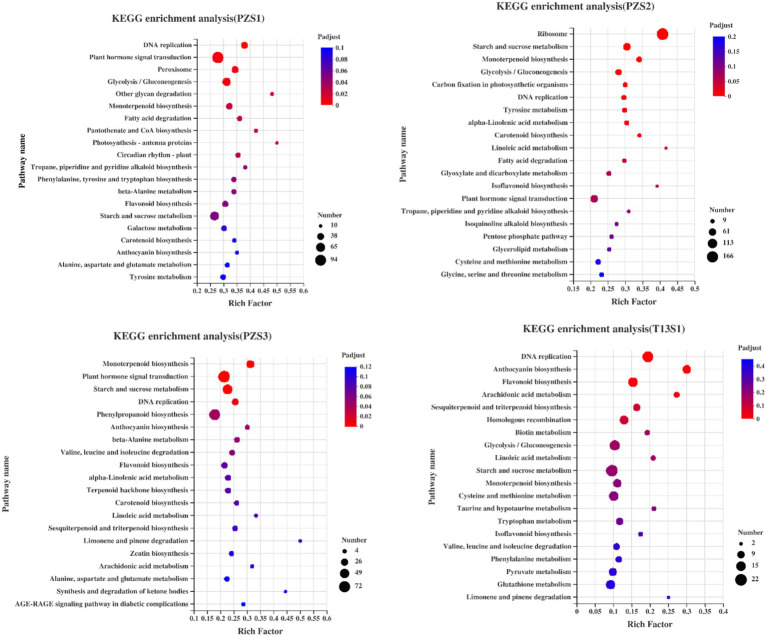
Kyoto Encyclopedia of Genes and Genomes (KEGG) analysis of all DEGs in four pairwise groups: PZS1H vs. PZS1D (PZS1), PZS2H vs. PZS2D (PZS2), PZS3H vs. PZS3D (PZS3), and T13S1H vs. T13S1D (T13S1). The circle size represents the number of DEGs detected in the KEGG pathway. The rich factor is the ratio of DEGs to the total background gene number in each pathway.

Next, KEGG enrichment among the DEGs was analyzed. As shown in Figure 3, the plant hormone signaling transduction pathway was identified in all of the PZ groups, and the peroxisome pathway was the top term in PZS1. However, these terms were not enriched in the top 20 terms in “T13.” These results demonstrated that plant hormone signaling or oxidative pathways might have important functions in phytoplasma differential resistance between PZ and T13.

**Figure 4:**
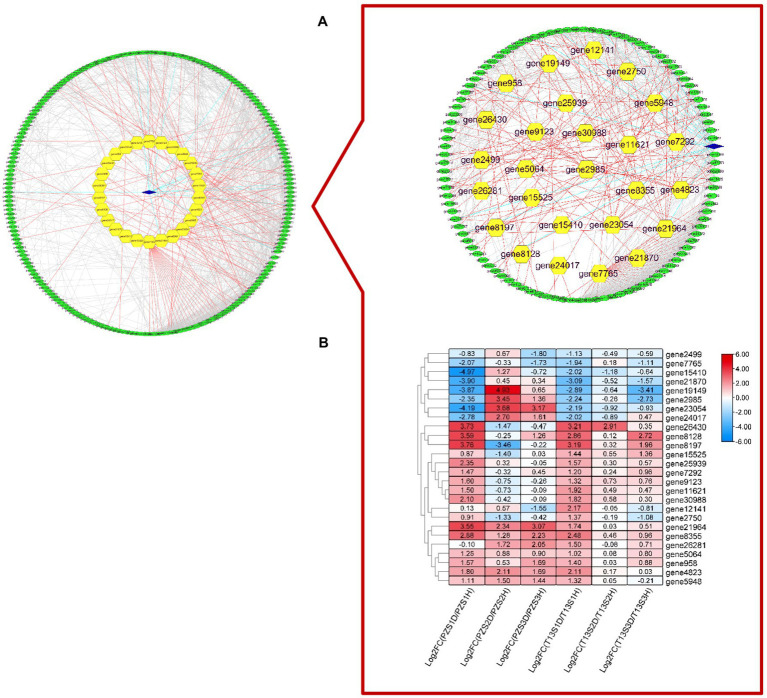
The protein–protein interaction prediction of 418 key DEGs that were mainly involved in hormone and oxidative processes with Gene Ontology (GO) and KEGG enrichment selection. **(A)** Protein–protein interaction prediction of 235 DEGs. **(B)** Heatmap of the expression level of the 26 key genes in six pairwise comparisons (T13S1H vs. T13S1D, T13S2H vs. T13S2D, T13S3H vs. T13S3D, PZS1H vs. PZS1D, PZS2H vs. PZS2D, and PZS3H vs. PZS3D) with log 2 Fold calculation. Green ovals represent DEGs expressed in “PZ,” blue diamonds represent DEGs expressed in “T13,” and yellow hexagons represent DEGs expressed in “T13” and “PZ.”

According to the KEGG analysis, 418 DEGs involved in seven biological processes, including but not limited to plant hormone signal transduction, peroxisome, and plant–pathogen interactions, were selected to perform protein–protein interaction network prediction. As shown in Figure 4A, 235 DEGs had a certain protein interaction relationship. Among these genes, only one gene (gene26951), which was described as serine/threonine-protein kinase SAPK3-like, was expressed in “T13,” while 26 DEGs were differentially expressed in both materials, and the rest were expressed in “PZ.” The 26 DEGs expressed in both materials were of considerable interest. As shown in Figure 4B, the expression patterns of 26 DEGs in “T13” and “PZ” in response to phytoplasma infection were different. Among them, the DEG genes involved in auxin synthesis and efflux, cytokinin, JA, calcium, MAPK, and ROS signaling were identified. For example, mitogen-activated protein kinase kinase 6 (gene24017) was highly induced in growth phases S2 and S3 in “PZ” but not in “T13.” Auxin transporter-like protein 2 (gene19149), which belongs to the AUXIN1/LIKE-AUX1 (AUX/LAX) gene family and encodes the major auxin influx carrier, was significantly upregulated in growth phase S2 in “PZ,” but downregulated in “T13.” TIFY 6B (gene11621), which belongs to the JAZ subgroup, could interact with MYC2 involved in JA signal transduction and was induced in growth phase S1 in both materials and downregulated in growth phases S2 and S3. Respiratory burst oxidase homologue protein B (gene4823) was highly induced in growth phases S1 to S3 in “PZ,” upregulated in growth phase S1 in “T13,” and downregulated in the other two growth phases in “T13.” These results demonstrated that hormone and ROS signaling, especially auxin, cytokinin, or JA, might contribute to the differential phytoplasma resistance between PZ and T13.

**Figure 5:**
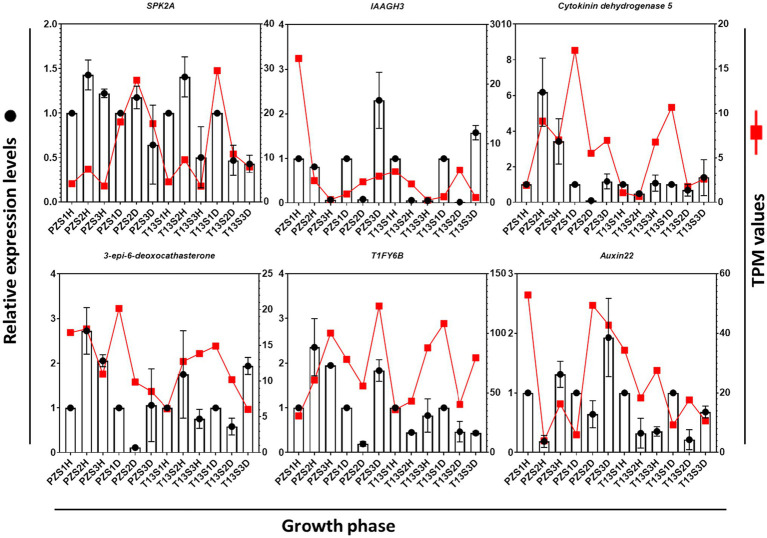
Comparison of the expression levels of six selected DEGs with transcripts per million (TPM) values. Quantitative real-time PCR (qRT–PCR) values represent the column chart (*y*-axis on the left), and transcriptome values are shown as red lines (*y*-axis on the right). The *x*-axis represents three growth phases in healthy and diseased “PZ” and “T13.”

To verify the transcriptome analysis of the above 26 DEGs, six of them involved in hormone signaling and oxidative pathways, including serine/threonine-protein kinase SRK2A-like (SRK2A), indole-3-acetic acid-amido synthetase GH3.17-like (IAAGH3), cytokinin dehydrogenase 5,3-epi-6-deoxocathasterone 23-monooxygenase, auxin-induced protein 22C (Auxin22), and protein TIFY 6B (TIFY6B), were selected for qRT–PCR analysis. As shown in Figure 5, the expression levels of these six genes were consistent with the changes in TPM values. For example, the expression level of *auxin22* was highly induced from the S1 to S3 growth phases in “PZ” diseased plants. The TPM values also increased at these growth phases in “PZ” diseased plants. These results demonstrated that our transcriptome data were reliable.

### ROS Accumulation Was Observed at the Recovery Growth Phase in “T13” but not in “PZ”

**Figure 6:**
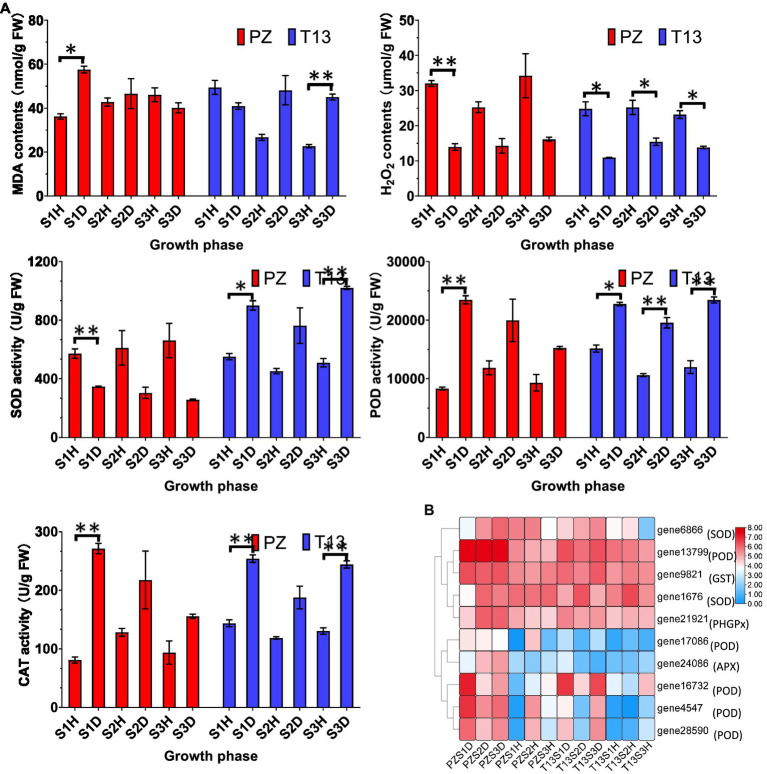
Physiological changes and heatmap of DEGs involved in reactive oxygen species (ROS) in PZ and T13 leaves infected by phytoplasma. **(A)** The levels of malondialdehyde (MDA) and hydrogen peroxide (H_2_O_2_) contents and superoxide dismutase (SOD), peroxidase (POD) and catalase (CAT) activities in “PZ” and “T13” leaves in three growth phases in healthy and diseased “PZ” and “T13” with three biological replications. Error bar represents SE (*n* = 3). **(B)** Heatmap of the expression level of key genes involved in the ROS pathway with Log2FC calculation. PZDS1 and T13DS1 represent the samples from the S1 growth phase of diseased “PZ” and “T13,” respectively. The other labels in the heatmap have the corresponding meanings as well as PZDS1 or T13DS1. *p < 0.05; and **p < 0.01.

High levels of ROS accumulation are toxic to plant cells, and proper accumulation functions are important in signal transduction. GO and KEGG enrichments indicated that the peroxisome pathway was the top term in PZS1 but not in T13S1. Thus, the contents of MDA and H_2_O_2_ and the activities of SOD, POD, and CAT were analyzed in both materials. As shown in Figure 6, the level of MDA was higher in the PZS1D growth phase than in the healthy plants, while it decreased in the T13S1D growth phase compared to the healthy plants. Then, with the recovered phenotype of “T13” diseased plants, the MDA contents increased in T13S2D and T13S3D compared to their healthy plants. However, the level of H_2_O_2_ was much lower in both diseased plants at the three growth phases after phytoplasma infection than in their healthy plants. There was a significant increase from the T13S1D to T13S2D growth phases and a decrease in T13S3D. Lower contents of H_2_O_2_ were detected under phytoplasma infection in both plants, and a significant increase in H_2_O_2_ at the recovery phase demonstrated that H_2_O_2_ could be an early signaling molecule to activate the defense system in “T13.” Furthermore, SOD activity increased in diseased “T13” plants but declined in diseased “PZ” plants at three growth phases compared to their healthy plants, while the activity of POD and CAT, which could catalyze H_2_O_2_ to H_2_O, showed higher levels in both diseased plants at the three growth phases. In addition, from our transcriptomic data, we observed that the expression levels of antioxidant genes enriched in GO terms, including PODs, SODs, ascorbate peroxidase (APX), glutathione peroxidase (GPX), and glutathione S-transferase (GST), were induced in both diseased plants compared to their healthy controls (Figure 6B). The expression level changes of these genes were consistent with the changes in SOD, POD, and CAT activities in both plants in response to phytoplasma infection, suggesting that the higher level of POD and CAT activity contributed to the lower generation of H_2_O_2,_ which could function as a signaling molecule against phytoplasma infection.

### A Higher Level of the Ratio of Zeatin to Auxin Contributed to JWB Susceptibility

**Figure 7:**
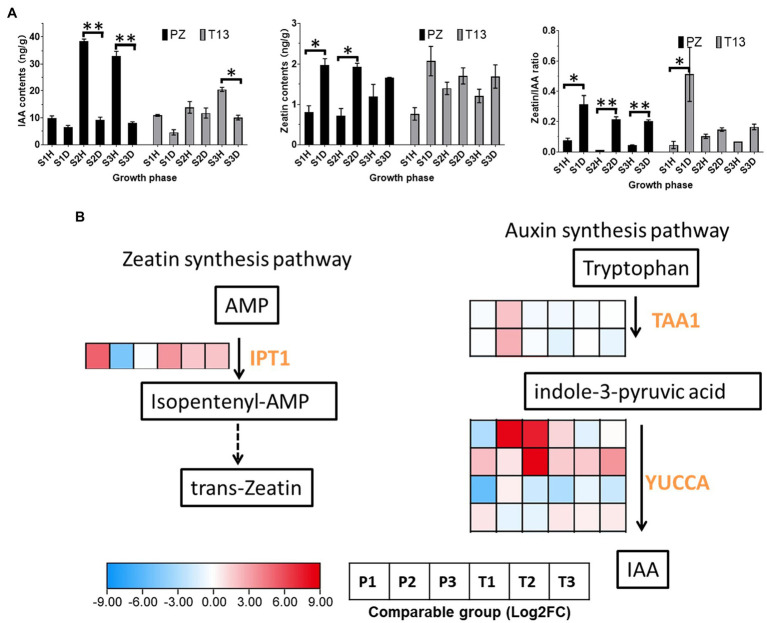
Auxin and cytokinin changes and heatmap of DEGs involved in their synthesis pathway in “PZ” and “T13” leaves infected by phytoplasma. **(A)** IAA and zeatin contents and the ratio of zeatin to IAA changes in three growth phases in healthy and diseased “PZ” and “T13” with three biological replications. Error bar represents SE (*n* = 3). **(B)** Heatmap of the expression level of key genes involved in IAA and the zeatin synthesis pathway with Log2FC calculation. P1 and T1 represent the Log2(disease/healthy) results at growth phase S1 in “PZ” and “T13,” respectively. P2 and P3 and T2 and T3 have the corresponding meanings, as well as P1 and T1, respectively. IPT, isopentenyl transferase; TAA, tryptophan amino transferase; and YUCCA, flavin monooxygenase gene. *p < 0.05; and **p < 0.01.

The contents of auxin and zeatin between phytoplasma-sensitive “PZ” and phytoplasma-tolerant “T13” were tested in the current study. Firstly, the contents of IAA showed lower levels in both diseased and healthy “PZ” and “T13” plants at the S1 growth phase. With the growth development, the content of IAA increased significantly in “PZ” healthy plants at S2 and S3 growth phases. Then serious witches’ broom symptoms occurred in “PZ” diseased plants at the S2 and S3 growth phases that no significant changes of IAA content at these phases. However, the levels of IAA increased significantly in “T13” plants from the S1 to S2 and S3 growth phase in both healthy and recovered plants, demonstrating that higher contents of IAA negatively regulated witches’ broom symptoms (Figure 7A). The levels of zeatin were significantly induced in “PZ” diseased plants at three growth phases compared to healthy plants. However, the contents of zeatin significantly increased in “T13” diseased plants and growth phase S1 compared to healthy plants. Then, with the recovery of “T13” in response to phytoplasma infection, the levels of zeatin in T13S2D and T13S3D were the same as those in healthy plants and lower than those at T13S1D, suggesting that higher levels of zeatin positively contributed to witches’ broom symptoms (Figure 7A). Finally, the ratio of zeatin to IAA was analyzed; it was significantly higher in “PZ” diseased plants and T13S1 diseased plants, while it remained at lower levels in healthy “PZ” and “T13” plants and recovered T13S2 and T13S3 plants (Figure 7A). The results showed that a higher ratio of zeatin to auxin contributed to witches’ broom symptoms.

Compared to the transcriptomic data, the expression level of the key enzyme isopentenyl transferase (IPT), which can catalyze adenosine monophosphate (AMP) to synthesize zeatin, was dogged out. As shown in Figure 7B, the expression level of *IPT* was highly induced in the S1 growth phase in both materials compared to their healthy plants and then decreased from S2 to S3. This finding was consistent with the changes in the zeatin contents (diseased/healthy) in both materials in all three growth phases. In the IAA synthesis pathway, tryptophan amino transferase (*TAA*) is the first enzyme that can catalyze tryptophan to synthesize indole-3-pyruvic acid (*IPA*). Then, the flavin monooxygenase (*YUC*) gene family can catalyze IPA to synthesize IAA. In our study, two *TAAs* had the same expression profiles, which were highly induced in the S2 growth phase in PZ, and four *YUCCAs* had different expression patterns. The first two TAAs were significantly induced in the S2 or S3 growth phase in PZ, but the last TAA was highly induced at S1 to S3 in “T13.” Considering the changes of IAA contents in both plants, the expression level of the last *YUCCA* was consistent with the changes of IAA contents which might be the key gene of the *YUC* gene family, which function in the synthesis of IAA in “PZ” and “T13.”

### JA and SA Accumulation Associated With Phytoplasma Infection

**Figure 8:**
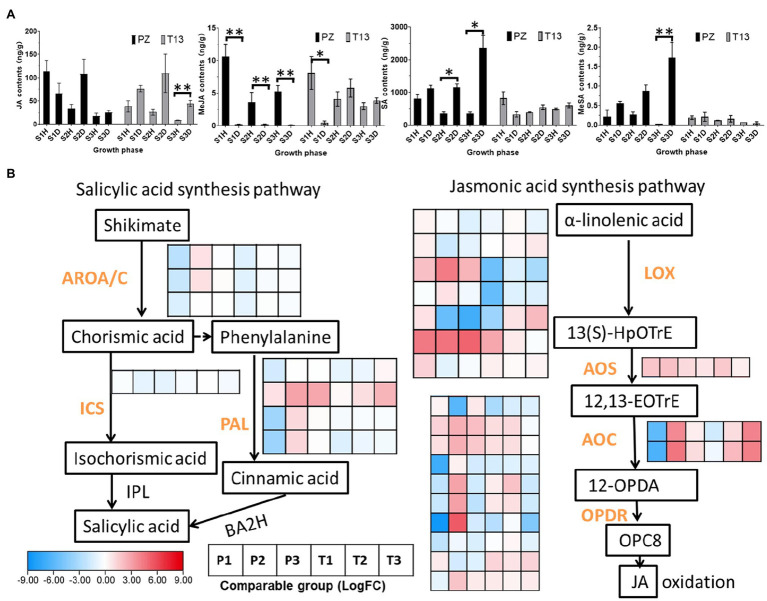
Jasmonic acid (JA), MeJA, salicylic acid (SA), and MeSA changes and heatmap of DEGs involved in their synthesis pathway in “PZ” and “T13” leaves infected by phytoplasma. **(A)** JA, MeJA, SA and MeSA changes in three growth phases in healthy and diseased “Pozao” and “T13” with three biological replications. Error bar represents SE (*n* = 3). **(B)** Heatmap of the expression level of key genes involved in the SA and JA synthesis pathways with Log2FC calculation. P1 and T1 represent the Log2(disease/healthy) results at growth phase S1 in “PZ” and “T13,” respectively. P2 and P3 and T2 and T3 have the corresponding meanings, as well as P1 and T1, respectively. ICS, isochorismate synthase; PAL, phenylalanine ammonia-lyase; LOX, lipoxygenase; AOS, allene oxide synthase; AOC, allene oxide cyclase; and OPDR, OPDA reductase. *p < 0.05; and **p < 0.01.

In addition to auxin and zeatin, JA and SA play pivotal roles in pathogen attack. Thus, the levels of JA, MeJA, SA, and MeSA after phytoplasma infection were detected in both materials. As shown in Figure 8A, the contents of JA accumulated in the first two growth phases in both diseased “PZ” and “T13” plants and decreased at S3. At the S2 growth phase, the JA concentration reached 110 ng/g in both diseased plants. However, the content of MeJA was tenfold lower than that of JA, and MeJA mainly accumulated in all healthy plants. For example, the MeJA content was above 4 ng/g in the three growth phases of “PZ” and “T13” healthy plants, while it was nearly undetectable in the three growth phases of “PZ” diseased plants and T13S1 diseased plants. However, when “T13” diseased plants recovered from the S1 to S2 growth phase, the contents of MeJA significantly increased from 1 to 6.5 ng/g and remained at the same level at the S3 growth phase, which was as high as in all the healthy plants. SA was highly accumulated in “PZ” diseased plants and increased from the S1 to S3 growth phases but decreased in “PZ” and “T13” healthy plants. In addition, compared to the “PZ” diseased plants, the contents of SA were lower in the “T13” diseased plants, possibly indicating that lower accumulation of SA was beneficial to phytoplasma resistance in the “T13” plants. The content of MeSA was much lower than that of SA in both materials, and it was highly accumulated in the three growth phases of “PZ”-diseased plants with the same level of change as that of SA.

Genes associated with JA and SA synthesis showed different expression patterns. In the JA pathway, the expression profiles of *LOX* (lipoxygenase), *AOS* (allene oxide synthase), *AOC* (allene oxide cyclase), and *OPDR* (OPDA reductase) changes determined the contents of JA. Among them, the two *AOCs* and the last *OPDR* gene were highly induced in the S2 growth phase of PZ and upregulated in the S2 to S3 phases in “T13,” consistent with the changes in JA contents at these growth phases in both materials. In the SA pathway, the expression of *BA2H* (benzoic acid-2-hydroxylase) and *IPL* (isochorismate pyruvate-lyase) was not significantly changed; thus, we identified upstream genes involved in SA synthesis, such as *ICS* (isochorismate synthase) and *PAL* (phenylalanine ammonia-lyase; Figure 8B). The expression of *ICS* showed no significant changes, but three *PALs* were highly induced in the S2 growth phase of “PZ” and not highly induced in the S2 growth phase of “T13,” which is consistent with the changes of SA contents.

### Exogenous Application of an SA Inhibitor Rescued the Symptoms of Jujube After Phytoplasma Infection

**Figure 9:**
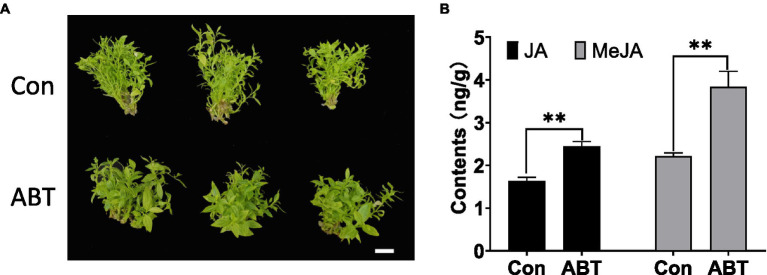
The phenotype of witches’ broom symptoms **(A)** and contents of JA and MeJA **(B)** in DH2 tissue culture after 1-aminobenzotriazole (ABT) treatment for 1 month. White bars are 1 cm. *p < 0.05; and **p < 0.01.

To gain insight into whether SA or JA could affect the symptoms of jujube in response to phytoplasma infection, the application of MeJA, SA (active molecules), or their inhibitors, such as ibuprofen, which can inhibit the synthesis of JA, and ABT, which can inhibit the activity of benzoic acid 2-hydroxylase (BA2H) to synthesize SA (Leon et al., 1995), was performed on DH2 (JWB tissue culture). As shown in Supplementary Figure S1, after 1 month of inoculation, regardless of the application of MeJA, ibuprofen, or SA to DH2 plants, they showed the same witches’ broom symptoms (especially small leaves) as the negative control. However, the DH2 plants treated with ABT showed different symptoms compared to those of the negative control (Figure 9A). Some branches of the plants showed healthy symptoms with greener and larger leaves, and they grew faster without yellow leaves. Furthermore, with ABT treatment in DH2, the content of SA should be decreased theoretically, so we would like to know how the content of JA and MeJA changed. As shown in Figure 9B, the contents of both JA and MeJA increased after ABT treatment compared to the control. These results demonstrated that exogenous application of an SA inhibitor rescued the symptoms of jujube after phytoplasma infection by decreasing the contents of SA and increasing the contents of JA and MeJA.

## Discussion

Phytoplasma disease has destructive effects in Chinese jujube. Here, we chose two cultivars, “PZ” and “T13,” with different phytoplasma resistance as plant materials to perform transcriptome and ROS hormone signaling studies, furthering our knowledge of the phytoplasma resistance mechanism, especially from the signal transduction and hormone crosstalk perspectives.

### ROS Accumulation Could Be an Early Signaling Molecule in Response to Phytoplasma

Signal transduction plays an important role in the plant defense system, including in the response to phytoplasma. In addition to calcium, ROS are one of the major signaling molecules against phytoplasmas (Musetti et al., 2004; Sanchez-Rojo et al., 2011; Patui et al., 2013). In many horticultural plants, such as apple, grapevine, peach, and apricot, the generation of ROS in response to phytoplasma has been widely observed (Gambino et al., 2013). Among ROS, H_2_O_2_ is considered the key signaling molecule that can diffuse into the cytosol as a signaling molecule involved in programmed cell death (Wan et al., 2021; Wang et al., 2021). As the location of phytoplasma in Chinese jujube was in the phloem (Xue et al., 2020), the production of H_2_O_2_ in phytoplasma-recovered apple plants was also localized in the phloem tissue, and higher level accumulation of H_2_O_2_ was detected, which could improve phytoplasma resistance, implying that H_2_O_2_ could function as a signal to activate the defense response under phytoplasma attack (Musetti et al., 2010; Torres, 2010). In our study, transcriptome analysis between “PZ” and “T13” under phytoplasma attack showed many DEGs involved in biological processes known to be associated with peroxisomes, demonstrating that ROS might function in response to phytoplasma disease. The measurement of ROS showed that the level of H_2_O_2_ was lower in both diseased plants at three growth phases after phytoplasma infection than in their healthy control plants. However, during the recovered phase in “T13” from the S1 to S2 phases, there was a significant increase in H_2_O_2_. In addition, when diseased “T13” became healthy from the S2 to S3 phases, the H_2_O_2_ content decreased. Changes in H_2_O_2_ were not observed in the phytoplasma-sensitive cultivar PZ. The same changes in H_2_O_2_ were found in the recovered apple plants infected with phytoplasma. Moreover, during the recovery, plasma membrane NAD(P)H peroxidases were continuously active to produce more H_2_O_2_ that could act indirectly as a signal molecule to facilitate the recovery process by inducing the disappearance of phytoplasma from the canopy (Patui et al., 2013). In addition, MDA is one of the final products of phospholipid peroxidation responsible for cell membrane damage, and an increase in lipid peroxidation under stress parallels an increased production of ROS (Sharma et al., 2012). In our study, with the recovered phenotype of “T13” diseased plants, the MDA contents increased in T13S2D and T13S3D compared to their healthy control plants, which was consistent with the changes in H_2_O_2_. Together, these results demonstrated that H_2_O_2_ could be an important signaling molecule that contributes to the phytoplasma resistance of “T13.”

### Crosstalk of JA and SA Accumulation Leads to Phytoplasma Resistance in “T13”

Plant hormones are small organic molecules involved in plant growth and development and participate in plant defense against pathogen attack (Shigenaga et al., 2017; Dermastia, 2019; Wasternack and Strnad, 2019). Among them, JA and SA are the critical hormones involved in plant immunity. JA is mainly involved in plant immunity against necrotrophic pathogens, insect attack, and wounding, while SA participates in the resistance response to biotrophic and hemibiotrophic pathogens (Howe and Jander, 2008; Bari and Jones, 2009). However, hormones do not function alone; they communicate together as complex networks of interactions, which is called hormone crosstalk, to respond to pathogen attack (Kohli et al., 2013). For example, SA is an important antagonist of the JA response (Koornneef et al., 2008). SA can suppress JA signaling through downregulation of JA-responsive gene expression in *Arabidopsis* (Gupta et al., 2000; Spoel et al., 2007). The properties of trade-off between JA and SA signaling have been found in numerous *Arabidopsis* studies (Leon-Reyes et al., 2010). In our study, JA significantly accumulated in diseased “PZ” plants, especially in the S2 growth phase, as well as in the S2 recovery phase in “T13,” while the JA content decreased at S3 in both materials. SA was highly induced in all three growth phases of “PZ” diseased plants and was especially significantly upregulated at S3 but accumulated at lower levels in “T13” diseased plants at the S1 and recovered S2 and S3 phases. Combining the crosstalk of JA and SA, the trade-off property of JA and SA could be observed in Chinese jujube in response to phytoplasma infection, in which higher accumulation of JA with lower accumulation SA contributed to phytoplasma resistance in “T13.” Furthermore, with exogenous application of MeJA, ibuprofen, or SA, DH2 plants could not inhibit witches’ broom symptoms, but exogenous application of an SA inhibitor rescued the symptoms of jujube after phytoplasma infection, demonstrating no JA accumulation, but lower SA contents conferred Chinese jujube “T13” resistance.

The function of JA in response to phytoplasma has been demonstrated in a Chinese jujube study by iTRAQ proteomics and RNA-seq transcriptomics (Ye et al., 2017; Wang et al., 2018). JA-related proteins and JA content in leaf samples increased under phytoplasma infection, while JA-related DEGs and DEPs were both downregulated, and JA content was slightly decreased during the JWB recovery process by tetracycline treatment (Wang et al., 2019). In combination with our study, we could further conclude that JA accumulation plays roles in defending against phytoplasma infection in Chinese jujube, but SA functioned more importantly in the recovery growth phase and finally contributed to defense resistance in “T13.” In other words, the trade-off of JA (high accumulation) and SA (lower accumulation) was positive for phytoplasma resistance in “T13.” However, the potential molecular mechanism by which SA regulates phytoplasma resistance in Chinese jujube should be further studied.

### Perspectives of Different Signal Transduction Pathways, Including ROS, JA, and SA, Between “PZ” and “T13” Against Phytoplasma

**Figure 10:**
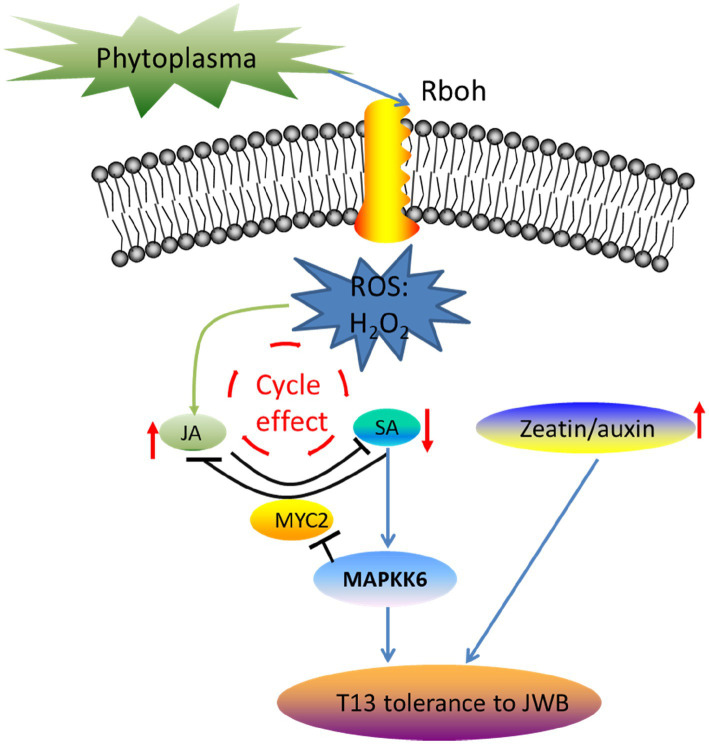
The working model of how Chinese jujube “T13” resisted phytoplasma infection. Phytoplasma infection induces rapid ROS generation, which may be the initial signaling molecule to amplify the accumulation of JA, which could be further antagonistic to SA accumulation to decrease its contents. In addition, the cycle effect between ROS and JA/SA functions to amplify signaling. Moreover, lower contents of SA could shut down the upregulation of *MAPKK6*, which could downregulate the expression level of *MYC2* to inhibit the JA signal and activate the expression of downstream genes to confirm phytoplasma resistance. Another pathway is that a higher level of the ratio of zeatin to auxin contributes to phytoplasma resistance. The red arrow indicates the increase or decrease in the corresponding hormones.

ROS produced by respiratory burst oxidase homologue (RboH) function as an important signaling molecule in the process of signal transduction in response to pathogen attack (Wang et al., 2021). The relationship between ROS signaling and hormones has been studied, demonstrating that they do not work in parallel but mutually affect each other. For example, an increase in ROS could be an important signal to amplify and mediate JA- and SA-induced defense responses to pathogens (Torres, 2010). The crosstalk between ROS and hormones in response to biotic and abiotic stresses has been widely studied (Devireddy et al., 2021). SA and ROS are involved in plant program cell death and disease resistance (Huysmans et al., 2017; Zhang et al., 2020). A mimic of JA-Ile virulence factor coronatine (COR), which is secreted by the Pst pathogen, could activate *MYC2* and *NAC019/055/072* to repress SA synthesis (Zheng et al., 2012). In addition, some key genes are involved in JA and SA signal transduction, such as mitogen-activated protein kinase (*MAPK*; Rodriguez et al., 2010), *MYC2*, and *PDF1.2* (Gatz, 2013). *MPK4* could positively regulate *GRX480*, which could specifically bind to *TGAs* that regulated the expression level of *PR1* in the SA signaling pathway, while *MPK4* could negatively regulate the expression level of *MYC2* in the JA signaling pathway (Wasternack and Song, 2017). Thus, *MYC2* and its upstream *MPK4* are the two key genes involved in the crosstalk between JA and SA signaling pathways, which coordinately regulate plant disease resistance (Yang et al., 2019). In our study, the core genes identified by transcriptome analysis showed that *TIFY6B* (gene11621), which belongs to the JAZ subgroup, interacted with *MYC2*, which is involved in JA signal transduction and was downregulated in “T13” diseased plants compared with “PZ.” Mitogen-activated protein kinase kinase 6 (*MAPKK6*, gene24017) was highly induced in “PZ” diseased plants but not in “T13” diseased plants. Combining these results with those of a previous study, we developed a model of how Chinese jujube “T13” resisted phytoplasma infection (Figure 10). First, rapid ROS generation could be the initial signaling molecule to amplify the accumulation of JA, which could be further antagonistic to SA accumulation to decrease its content. In addition, the cycle effect between ROS and JA/SA functions to amplify signaling. Moreover, lower contents of SA could shut down the upregulation of *MAPKK6*, which could downregulate the expression level of *MYC2* to inhibit JA signaling and activate the expression of key downstream genes to confer phytoplasma resistance. A higher level of the zeatin-to-auxin ratio contributing to phytoplasma resistance represents another pathway.

## Data Availability Statement

The data sets presented in this study can be found in online repositories. The names of the repository/repositories and accession number(s) can be found at: NCBI-PRJNA798569.

## Author Contributions

ZL, LixW, and ML planned and designed the study. LD and JZ assisted with the experiments. LixW, SL, MG, and LinW performed the experiments and analyzed the data. LihW, LixW, and ZL performed or supervised bioinformatic analysis of the RNA-Seq data and wrote the manuscript. ZL and ML provided guidance throughout the study. YW also performed the experiment. All authors contributed to the article and approved the submitted version.

## Funding

This work was supported by the Natural Science Foundation of Hebei Province (C2020204082); Hebei Province fund for Oversea Talent (C20210114); Green Channel Fund of Hebei Province Natural Science Foundation (C2019204308); Young Talent Project of Hebei Agricultural University Foundation (grant number YJ201853); National Key Research and Development Project (2019YFD1001605); Subsidy Funds for Hebei Jujube Industry Technology Research Institute after Operation Performance (205676155H); The Science and Technology Research Project of University in Hebei Province (QN2020205); and Science and Technology Research and Development Plan Project of Handan (19422011008-49).

## Conflict of Interest

The authors declare that the research was conducted in the absence of any commercial or financial relationships that could be construed as a potential conflict of interest.

## Publisher’s Note

All claims expressed in this article are solely those of the authors and do not necessarily represent those of their affiliated organizations, or those of the publisher, the editors and the reviewers. Any product that may be evaluated in this article, or claim that may be made by its manufacturer, is not guaranteed or endorsed by the publisher.

## Supplementary Material

The Supplementary Material for this article can be found online at: https://www.frontiersin.org/articles/10.3389/fmicb.2022.800762/full#supplementary-material

## Footnotes

^1^ https://github.com/jstjohn/SeqPrep

^2^ https://github.com/najoshi/sickle

^3^ http://tophat.cbcb.umd.edu/

^4^ https://www.ncbi.nlm.nih.gov/genome/?term=Ziziphus+jujuba

^5^ http://deweylab.biostat.wisc.edu/rsem/

^6^ http://www.majorbio.com/

^7^ https://github.com/tanghaibao/Goatools

^8^ http://kobas.cbi.pku.edu.cn/home.do

^9^ https://string-db.org/
