# Supplementary material for: The Crosstalk of the Salicylic Acid and Jasmonic Acid Signaling Pathways Contributed to Different Resistance to Phytoplasma Infection Between the Two Genotypes in Chinese Jujube
Source: Front Microbiol. 2022 Mar 18;13:800762. doi: 10.3389/fmicb.2022.800762 (PMC8971994; doi:10.3389/fmicb.2022.800762)
Supplement: Supplementary file 1 [file Data_Sheet_1.ZIP › Figure S1.pptx]

## Slide 1
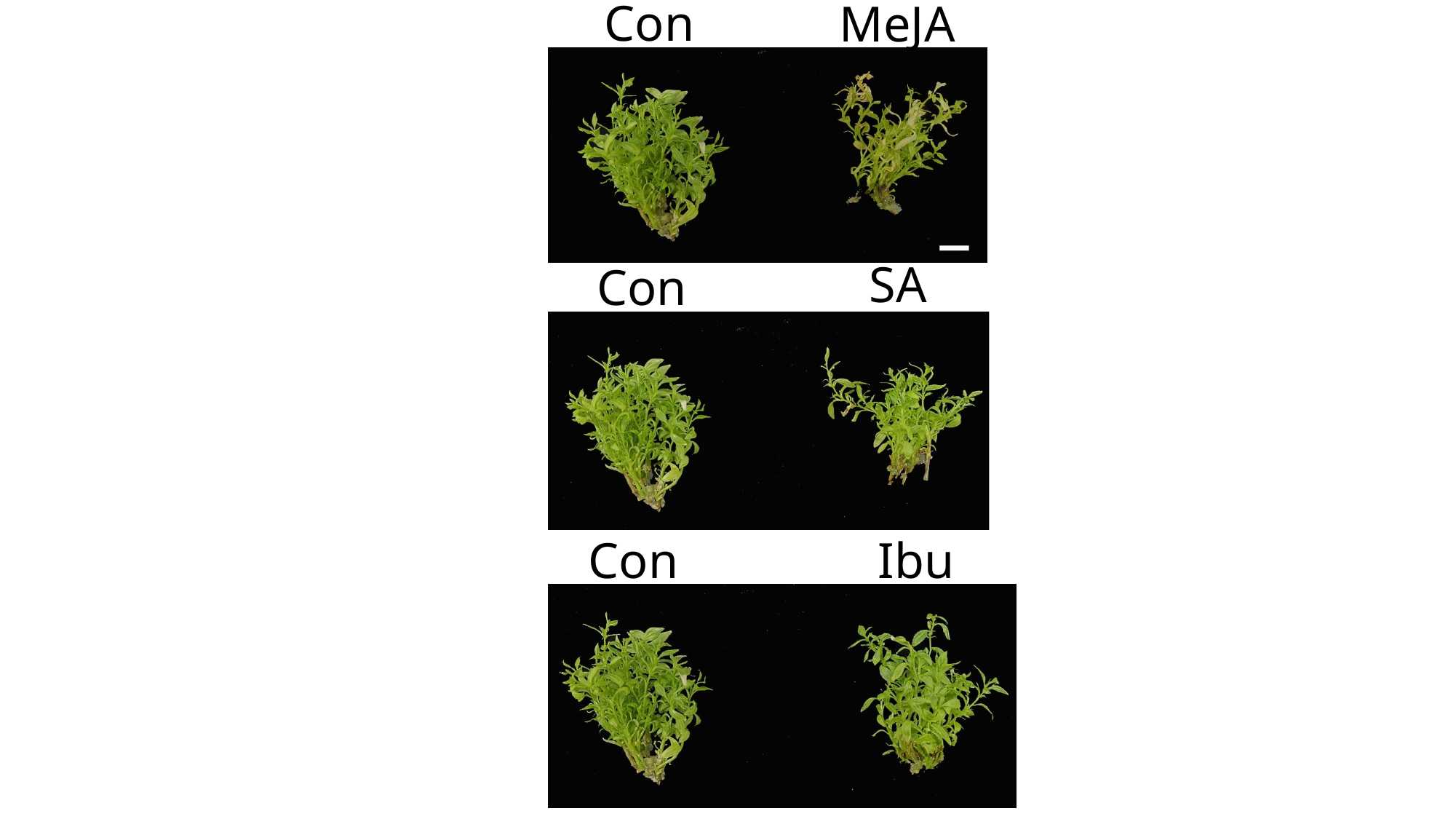

Con
MeJA
SA
Con
Ibu
Con

## Slide 2
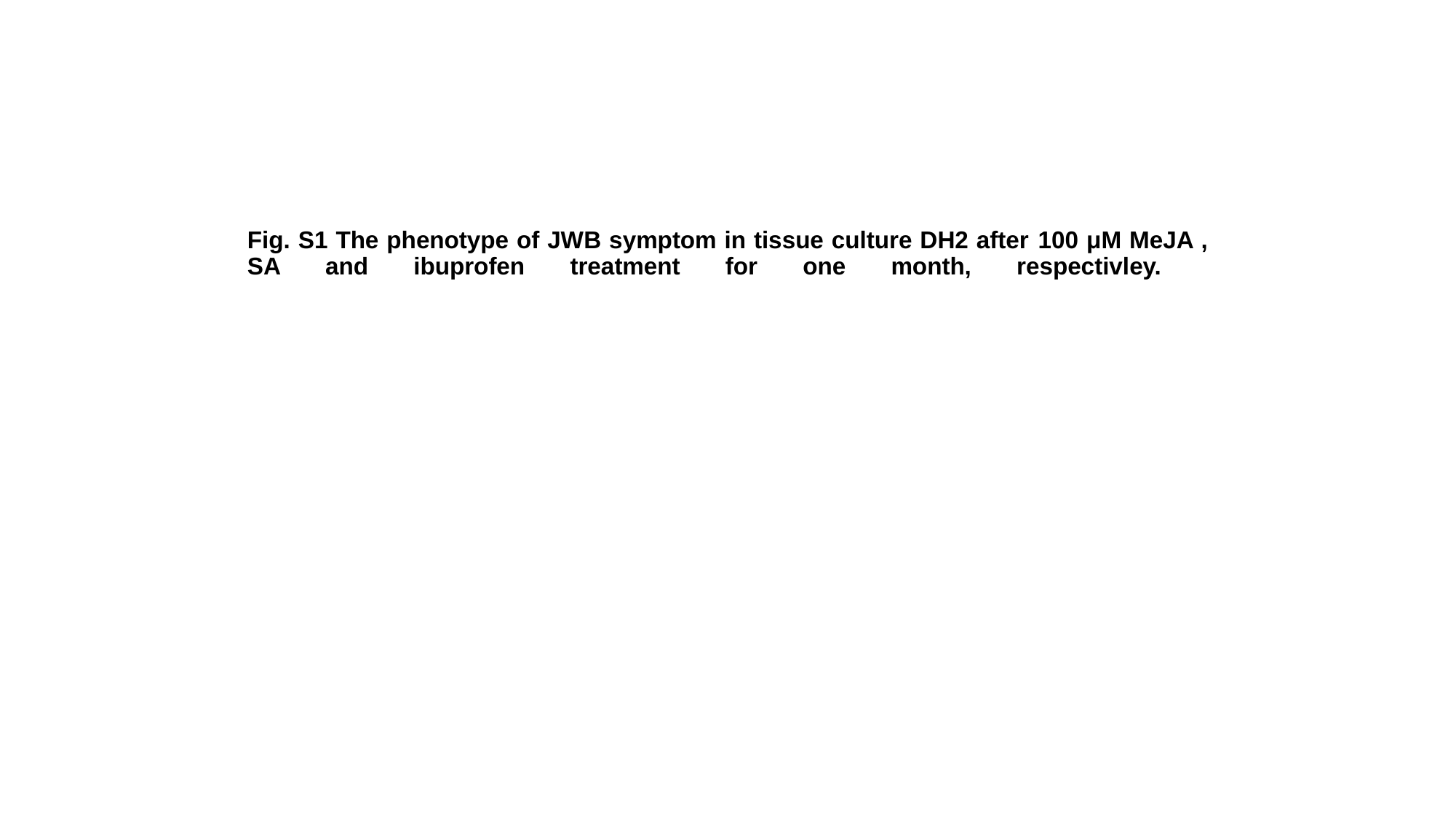

# Fig. S1 The phenotype of JWB symptom in tissue culture DH2 after 100 μM MeJA , SA and ibuprofen treatment for one month, respectivley.
